# Phosphorylation and oligomerization of α-synuclein associated with GSK-3β activation in the rTg4510 mouse model of tauopathy

**DOI:** 10.1186/s40478-020-00969-8

**Published:** 2020-06-19

**Authors:** Yuta Takaichi, James K. Chambers, Hiroyuki Inoue, Yasuhisa Ano, Akihiko Takashima, Hiroyuki Nakayama, Kazuyuki Uchida

**Affiliations:** 1 Laboratory of Veterinary Pathology, Graduate School of Agricultural and Life Sciences, The University of Tokyo, Tokyo, 113-8657 Japan; 2 Research Laboratories for Health Science & Food Technologies and the Central Laboratories for Key Technologies, Kirin Company Ltd., Kanagawa, Japan; 3 Department of Life Science, Faculty of Science, Gakushuin University, Tokyo, Japan

**Keywords:** α-Synuclein, Tau, Phosphorylation, Oligomerization, GSK-3β, rTg4510

## Abstract

Neurodegenerative diseases are characterized by the accumulation of specific phosphorylated protein aggregates in the brain, such as hyperphosphorylated tau (hp-tau) in tauopathies and phosphorylated α-synuclein (p-αSyn) in α-synucleinopathies. The simultaneous accumulation of different proteins is a common event in many neurodegenerative diseases. We herein describe the detection of the phosphorylation and dimerization of αSyn and activation of GSK-3β, a major kinase known to phosphorylate tau and αSyn, in the brains of rTg4510 mice that overexpress human P301L mutant tau. Immunohistochemistry showed p-αSyn aggregates in rTg4510 mice, which were suppressed by doxycycline-mediated decreases in mutant tau expression levels. A semi-quantitative analysis revealed a regional correlation between hp-tau and p-αSyn accumulation in rTg4510 mice. Furthermore, proteinase K-resistant αSyn aggregates were found in the region with excessive hp-tau accumulation in rTg4510 mice, and these aggregates were morphologically different from proteinase K-susceptible p-αSyn aggregates. Western blotting revealed decreases in p-αSyn monomers in TBS- and sarkosyl-soluble fractions and increases in ubiquitinated p-αSyn dimers in sarkosyl-soluble and insoluble fractions in rTg4510 mice. Furthermore, an activated form of GSK-3β was immunohistochemically detected within cells containing both hp-tau and p-αSyn aggregates. A semi-quantitative analysis revealed that increased GSK-3β activity strongly correlated with hp-tau and p-αSyn accumulation in rTg4510 mice. Collectively, the present results suggest that the overexpression of human P301L mutant tau promoted the phosphorylation and dimerization of endogenous αSyn by activating GSK-3β in rTg4510 mice. This synergic effect between tau, αSyn, and GSK-3β may be involved in the pathophysiology of several neurodegenerative diseases that show the accumulation of both tau and αSyn.

## Introduction

The accumulation of specific phosphorylated protein aggregates and neuronal loss in the central nervous system are hallmarks of several neurodegenerative diseases. The current pathological classification of neurodegenerative diseases is based on the proteins that accumulate. Intracytoplasmic hyperphosphorylated tau (hp-tau) aggregates are pathognomonic in tauopathies such as Alzheimer’s disease (AD), frontotemporal dementia with Parkinsonism linked to chromosome 17 (FTDP-17), and progressive supranuclear palsy (PSP), while phosphorylated α-synuclein (p-αSyn) aggregates are involved in α-synucleinopathies including Parkinson’s disease (PD), dementia with Lewy bodies (DLB), and multiple system atrophy (MSA) [50].

Tau and αSyn are both natively unfolded soluble proteins that are minimally phosphorylated in the normal adult brain. However, they undergo conformational changes, such as phosphorylation, in pathological states, which subsequently leads to oligomerization. They may also become ubiquitinated and form intracytoplasmic insoluble filamentous aggregates [30, 57]. In AD, tau proteins are hyperphosphorylated at various sites and form ultrastructural paired helical filaments (PHFs), resulting in neurofibrillary tangles (NFTs) [1]. Recent data has brought the total number of identified phosphorylation sites on tau from AD brain to 45, which represents more than half of the total of 85 phosphorylatable residues in tau. Though attempts to identify AD-specific phosphorylation sites on tau have yet to yield conclusive results, anti-hp-tau antibodies, which recognize tau phosphorylated at specific sites such as serine 199, 202, and 396 and threonine 205 positions, are widely used to detect AD pathology [19]. In α-synucleinopathies, αSyn proteins are mainly phosphorylated at the serine 129 position and form intracytoplasmic inclusions called Lewy bodies (LBs) [2]. The phosphorylation of tau and αSyn is controlled by several kinases and phosphatases [33, 57]. Glycogen synthase kinase-3β (GSK-3β) and protein phosphatase-2A (PP2A) are involved in the phosphorylation processes of both tau and αSyn [1, 10]. Postmortem studies on AD and PD brains revealed the overactivation of GSK-3β and inactivation of PP2A [10, 27, 55]. The levels of polo-like kinase 2 (PLK2), a major phosphorylation enzyme for αSyn, but not for tau, were found to be elevated in the brains of AD patients [35]. Recent studies proposed a seeding hypothesis in which small amounts of misfolded proteins act as seeds that initiate the recruitment of their soluble counterparts into fibrils and the cell-to-cell transmission of protein aggregates [30, 34]. The spread of hp-tau and p-αSyn accumulation closely correlates with disease progression; therefore, the distribution of hp-tau and p-αSyn is important for identifying the disease stages of sporadic AD and PD, respectively [30, 46]. Furthermore, an oligomer hypothesis has been proposed in α-synucleinopathies [43]. Under normal conditions, αSyn molecules are unfolded soluble monomers or tetramers [3], but in pathological states these molecules are phosphorylated, have a propensity for folding and forming insoluble oligomers, and through protofibrils become mature fibrils, the main component of LBs [29]. Accumulating evidence suggests that the toxic form of αSyn is oligomers, rather than mature fibrils, which induce neuronal dysfunction and cell death [12].

The co-deposition of different pathological protein aggregates is common in the brains of individuals with neurodegenerative diseases [31, 39, 50]. Intracellular p-αSyn aggregates are frequently found together with hp-tau accumulation in tauopathies such as AD [24], PSP [54], and FTD [58]. Studies using antibodies against αSyn detected LBs in the amygdala of more than 60% of familial and sporadic AD cases [32]. Conversely, hp-tau aggregates have been found in more than 50% of α-synucleinopathy cases, including cases of PD, DLB, and MSA [20]. Clinical and postmortem studies revealed that cases of AD with LBs were associated with faster and more severe cognitive decline as well as accelerated mortality than AD cases without LBs [28, 42]. Filamentous aggregates composed of one protein (tau or αSyn) in the brain have also been suggested to directly promote the aggregation of other proteins through a process called cross-seeding [17, 31].

We recently detected p-αSyn aggregates in a tauopathy mouse model (rTg4510 mice) that overexpresses human P301L mutant tau, and the accumulation of hp-tau and p-αSyn increased in an age-dependent manner [52]. In the present study, we examined the relationship between the accumulation of hp-tau and p-αSyn in rTg4510 mice by suppressing the expression levels of mutant tau using doxycycline. The results obtained suggested that the accumulation of p-αSyn in rTg4510 mice was dependent on the extent of hp-tau accumulation. We also revealed decreases in p-αSyn monomers in TBS- and sarkosyl-soluble fractions and increases in ubiquitinated p-αSyn dimers in sarkosyl-soluble and insoluble fractions, and the activation of GSK-3β in rTg4510 mice. We herein report the pathological findings of α-synucleinopathy in the brains of rTg4510 mice and discuss the relationship between hp-tau and p-αSyn accumulation.

## Materials and methods

### Animals

A transgenic model for human tauopathy, rTg4510 mice, and control FVB/N-C57BL/6 J mice were used (https://www.alzforum.org/research-models/rtgtaup301l4510). These transgenic mice overexpress human tau, which contains the frontotemporal dementia-associated P301L mutation; tau expression may be suppressed by doxycycline. Regarding the expression of mutant tau in rTg4510 mice, the mutated gene, which is located downstream of a tetracycline-operon-responsive element, must be co-expressed with an activator transgene, which consists of the tet-off open reading frame located downstream of Ca^2+^-calmodulin kinase II promoter elements, resulting in human P301L tau overexpression restricted to forebrain structures, which characterized with tau pathology in the form of argyrophilic tangle-like inclusions in the cerebral cortex and hippocampus, and behavioral impairments by 3 months of age [49].

rTg4510 mice (transgenic for both a tau responder transgene and an activator transgene) and littermate wild-type control mice that do not express tau (lacking either the tau responder transgene or the activator transgene) were maintained in an experimental facility at the University of Tokyo. Mice were housed in a cage with free access to a standard diet (MS food, Oriental Yeast, Tokyo, Japan). To suppress CamkIIa-tTA-driven human tau transgene expression, rTg4510 mice were fed a diet containing 200 mg/kg doxycycline (5TP7, TestDiet, St. Louis, MO) ad libitum from 2.5 to 10 months of age (*n* = 10). The remaining mice were fed a standard diet without doxycycline (rTg4510, *n* = 11; wild-type control, n = 11). Transgenic mice were euthanized at 10 months of age, and the brains were collected. Experiments were approved by the Institutional Animal Care and Use Committee of the Graduate School of Agricultural and Life Science at the University of Tokyo (Approval No. L18–042).

### Histopathology

Brain tissue samples from the cerebrum and cerebellum were fixed in 10% neutral-buffered formalin, routinely processed, and embedded in paraffin wax. Formalin-fixed and paraffin-embedded tissues were cut into 2- or 8 μm-thick serial sections. Deparaffinized sections were then stained with hematoxylin and eosin (HE).

### Immunohistochemistry

Consecutive sections were stained using an immunoenzyme technique. After deparaffinization and rehydration, antigen retrieval was performed via heating to detect hp-tau, αSyn, GSK-3β, and p-GSK-3β (Tyr216). A formic acid treatment was used to detect p-αSyn. To deactivate endogenous peroxidase, sections were immersed in 1% hydrogen peroxide in methanol for 5 min. To avoid non-specific binding of the antibody, sections were immersed in 8% skim milk in Tris-buffered saline (TBS). The following primary antibodies were used: mouse anti-hp-tau (clone AT8, 1:500, Thermo Scientific, Rockford, IL), rabbit anti-αSyn (clone D37A6, 1:500, Cell Signaling Technology, Beverly, MA), rabbit anti-GSK-3β (clone 27C10, 1:100, Cell Signaling Technology), rabbit anti-p-GSK-3β (Tyr216) (1:100, Novus Biologicals, Centennial, CO), rabbit anti-p-αSyn (clone D1R1R, 1:500, Cell Signaling Technology), mouse anti-p-αSyn (clone 81A, 1:500, Abcam, Cambridge, UK), and rabbit anti-p-αSyn (clone EP1536Y, 1:500, Abcam). After an incubation with each primary antibody at 4 °C overnight, immunolabeled antigens were visualized using the Dako EnVision+ System (Dako, Glostrup, Denmark) with 0.02% 3′3-diaminobenzidine plus 0.01% hydrogen peroxide as a chromogen. For semiquantitative analysis, we used rTg4510 mice fed the standard (*n* = 11) or doxycycline diet (*n* = 10). To evaluate hp-tau and p-αSyn deposition and the activation of GSK-3β, the numbers of hp-tau (clone AT8)-, p-αSyn (clone D1R1R)-, and p-GSK-3β (Tyr216)-positive cells were counted, respectively. We counted the number of cells with intracytoplasmic grains that were stained positive with each of the antibodies. In each region, we counted three different areas (0.14mm^2^ each), and the average was used for the number of positive cells in the region of the mice.

### Proteinase K digestion

To detect proteinase K-resistant αSyn, after antigen retrieval, sections were treated with 50 μg/ml proteinase K (Wako, Osaka, Japan) in Tris HCl buffer containing 10 mM Tris-HCl (pH 7.8), 100 mM NaCl, and 0.1% Nonidet-P40 at 37 °C for 3 min. Sections were then subjected to immunohistochemical processing using a rabbit anti-αSyn antibody (clone D37A6, 1:500).

### Double-labeling immunofluorescence

To detect the spatial and temporal distribution of hp-tau, p-αSyn, and p-GSK-3β (Tyr216), we performed double-labeling immunofluorescence. After an incubation with each of the primary antibodies at 4 °C overnight, sections were washed with TBS, incubated with the corresponding secondary antibody at room temperature for 1 h, and then mounted with Vectashield (H-1500, Vector Laboratories, Burlingame, CA). The following primary antibodies were used: mouse anti-hp-tau (clone AT8, 1:100), mouse anti-p-αSyn (clone 81A, 1:100), and rabbit anti-p-GSK-3β (Tyr216) (1:100). The following secondary antibodies were used: Alexa Fluor 594-conjugated goat anti-mouse IgG (1:100, Invitrogen, Eugene, OR), Alexa Fluor 488-conjugated goat anti-rabbit IgG (1:100, Life Technologies, Eugene, OR), Alexa Fluor 594-conjugated goat anti-rabbit IgG (1:100, Life Technologies), and Alexa Fluor 488-conjugated goat anti-mouse IgG (1:100, Invitrogen). Fluorescent reaction products were optimally visualized using an argon ion laser in a Carl Zeiss LSM700 Confocal Laser Scanning Microscope (Carl Zeiss, Tokyo, Japan).

### Protein extraction

Regarding Western blotting, tissue samples from the hindbrain were homogenized in 7.5 volumes of TBS buffer containing 50 mM Tris (pH 7.4), 150 mM NaCl, 1 mM EGTA, and 1 mM EDTA. After centrifugation at 23,000 rpm at 4 °C for 15 min, supernatants were collected as TBS-soluble fractions. Pellets were rehomogenized in 7.5 volumes of sucrose buffer containing 0.32 M sucrose, 10 mM Tris/HCl (pH 7.4), 1 mM EGTA, and 0.8 M NaCl and centrifuged as described above. Supernatants were collected and incubated with sarkosyl (Wako, 1% final concentration) at 37 °C for 1 h, followed by centrifugation at 60,000 rpm at 4 °C for 30 min, and then collected as sarkosyl-soluble fractions. Pellets were resuspended in TBS buffer to a volume equivalent to the wet weight of the original tissue (sarkosyl-insoluble fractions).

### Western blotting

Protein samples were dissolved in Laemmli Sample Buffer (SB), which included 2-mercaptoethanol, and were then boiled for 10 min. Proteins dissolved in Laemmli SB were separated using a 5–20% gradient polyacrylamide gel (ATTO, Tokyo, Japan) and then transferred to a nitrocellulose membrane with a pore size of 0.20 μm (GE Healthcare Bio-Sciences AB, Uppsala, Sweden). Non-specific binding was blocked by a treatment with 1% skim milk for 60 min or Blocking One P (Nacalai Tesque, Kyoto, Japan) for 3 h. Membranes were probed with the following antibodies at 4 °C overnight: mouse anti-hp-tau (clone AT8, 1:500), rabbit anti-p-αSyn (clone D1R1R, 1:500), rabbit anti-GSK-3β (clone 27C10, 1:1000), rabbit anti-p-GSK-3β (Tyr216) (1:500), rabbit anti-PLK2 (1:500, Thermo Fisher Scientific, Waltham, MA), rabbit anti-PP2A-C (1:1000, Cell Signaling Technology), rabbit anti-p-PP2A-C (Tyr307) (clone F8, 1:500, Santa Cruz Biotechnology, Dallas, TX), and horseradish peroxidase (HRP)-conjugated mouse anti-β-actin (clone 8H10D10, 1:5000, Cell Signaling Technology). After washing the membranes with TBS containing Tween 20, a HRP-conjugated sheep anti-rabbit IgG antibody (1:5000, GE Healthcare UK, Little Chalfont, UK) or HRP-conjugated sheep anti-mouse IgG antibody (1:5000 GE Healthcare UK) was applied. Blots were developed using ECL Select Western Blotting Detection Reagent (GE Healthcare Bio-Sciences AB). Immunoreactive bands were detected using the ChemiDoc XRS+ System (Bio-Rad Laboratories, Hercules, CA).

### Statistical analysis

Data are shown as the mean ± standard deviation (SD). Comparisons of means between rTg4510 and control mice were performed using a one-way analysis of variance (ANOVA) followed by Tukey’s multiple comparison tests. Relationships between the amounts of hp-tau and p-αSyn that accumulated and activation of GSK-3β were examined using Spearman’s rank correlation. A *p* value < 0.05 was considered to be significant.

## Results

### Decreased hp-tau and p-αSyn deposition in doxycycline-treated rTg4510 mice

**Fig. 1:**
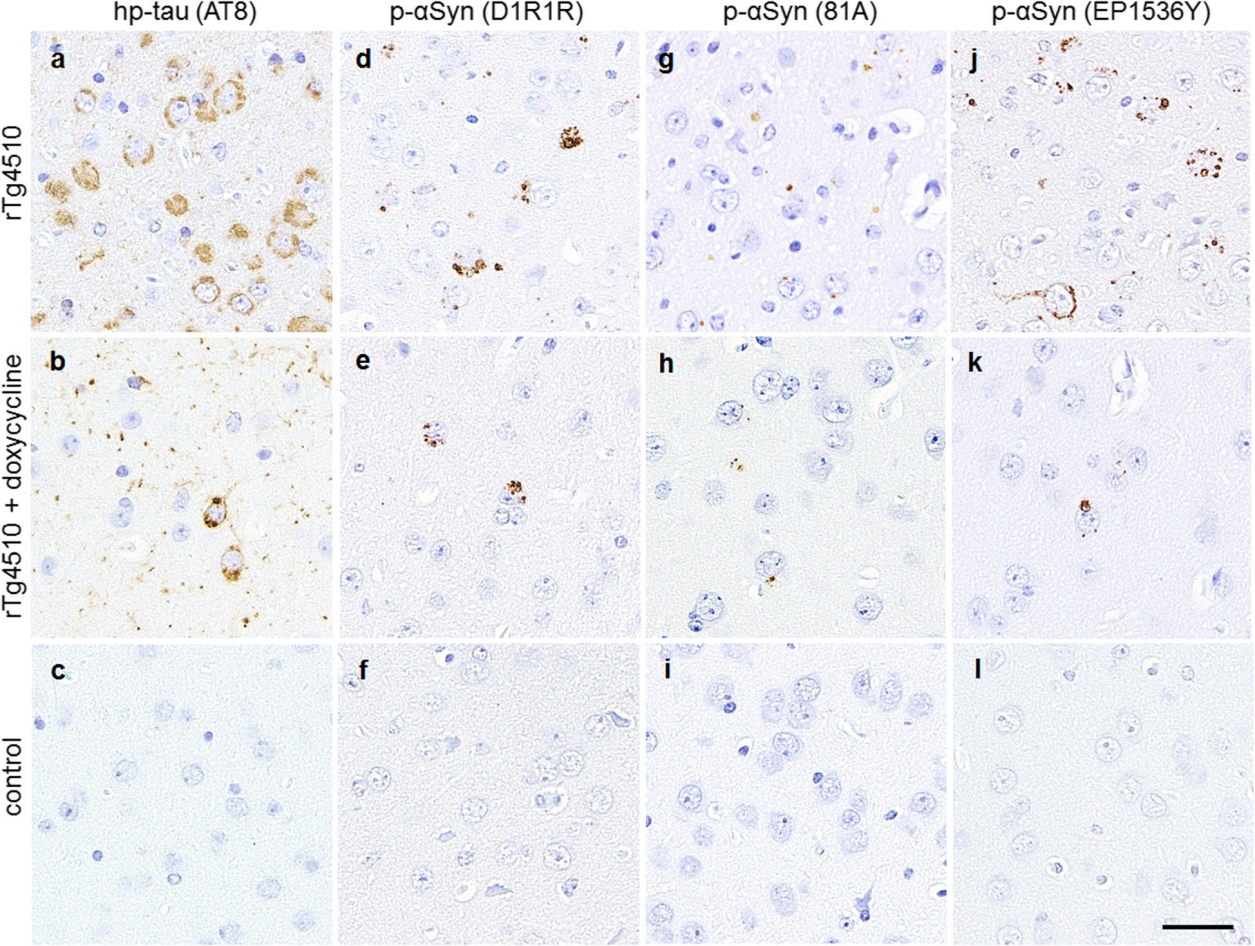
Immunohistochemistry for hp-tau and p-αSyn in the amygdala of rTg4510 mice fed the standard or doxycycline diet and control mice. rTg4510 mice developed numerous hp-tau-positive aggregates stained with the anti-hp-tau antibody AT8 (**a, b**), while control mice did not (**c**). rTg4510 mice also developed numerous, p-αSyn-positive aggregates stained with the anti-p-αSyn antibody D1R1R (**d, e**), 81A (**g**, **h**), and EP1536Y (**j, k**), while control mice did not (**f, i, l**). Scale bar: 20 μm (**a-l**)

**Fig. 2:**
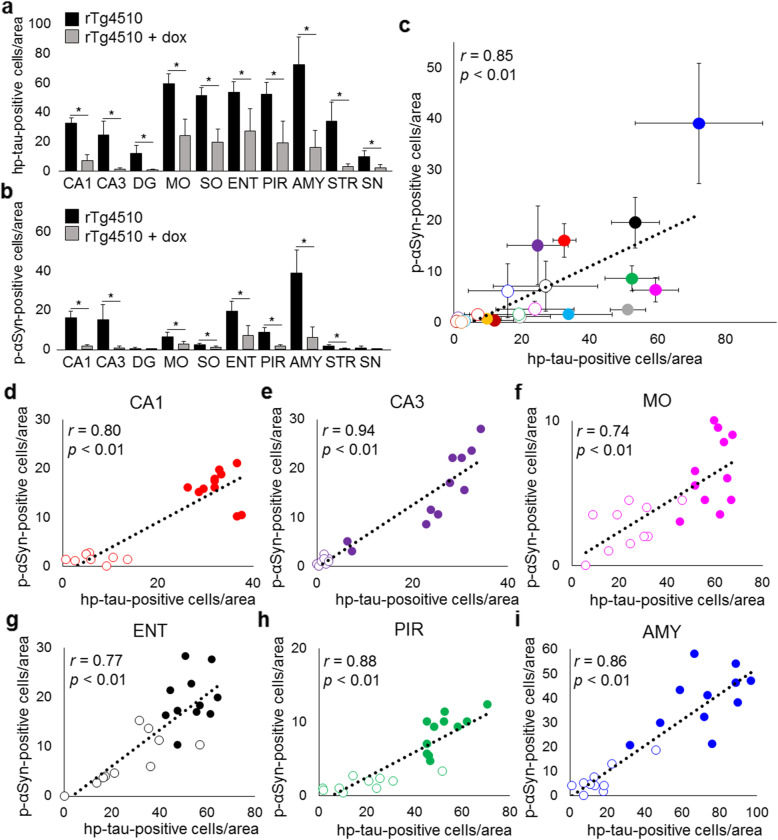
Semi-quantitative analysis of hp-tau and p-αSyn accumulation in 10 regions: the hippocampal CA1 and CA3 areas, dentate gyrus (DG), motor area (MO), somatosensory area (SO), entorhinal cortex (ENT), piriform cortex (PIR), amygdala (AMY), striatum (STR), and substantia nigra (SN), in rTg4510 mice fed the standard (*n* = 11) or doxycycline (dox) diet (*n* = 10). The accumulation of hp-tau was decreased in all regions studied in rTg4510 mice fed the dox diet (*p < 0.01, **a** The accumulation of hp-tau was decreased in CA1, CA3, MO, SO, ENT, PIR, AMY, and STR in rTg4510 mice fed the dox diet (*p < 0.01, **b**). Positive correlations were observed between the average accumulation of hp-tau and p-αSyn in each region (*r* = 0.85, *p* < 0.01, **c**), and between hp-tau and p-αSyn accumulation in CA1 (*r* = 0.80, *p* < 0.01, **d**), CA3(*r* = 0.94, *p* < 0.01, **e**), MO (*r* = 0.74, *p* < 0.01, **f**), ENT (*r* = 0.77, *p* < 0.01, **g**), PIR (*r* = 0.88, *p* < 0.01, **h**), and AMY (*r* = 0.86, *p* < 0.01, **i**) in rTg4510 mice. Colors: red, CA1; purple, CA3; deep red, DG; pink, MO; gray; SO; black, ENT; green; PIR; blue, AMY; light blue, STR; orange, SN. Diagrams: circular dot, rTg4510 mice fed the control diet; circle, rTg4510 mice fed the dox diet

We previously reported age-dependent increases in hp-tau and p-αSyn deposition in rTg4510 mice [52]. In the present study, the deposition of hp-tau and p-αSyn in doxycycline-treated rTg4510 mice was examined. rTg4510 mice fed the standard or doxycycline diet developed hp-tau aggregates in the neuronal cytoplasm and neurites of the cerebrum (Fig. 1a, b, Supplementary Fig. 1). Mice also developed p-αSyn-positive aggregates in the hippocampal CA1 and CA3 areas, dentate gyrus, motor area, somatosensory area, entorhinal cortex, piriform cortex, and amygdala (Fig. 1d, e, g, h, j, k, Supplementary Fig. 1), which corresponded to the distribution of hp-tau aggregates. However, there were no hp-tau or p-αSyn aggregates in the brains of control mice (Fig. 1c, f, i, l). To elucidate the relationship between the distribution of hp-tau and p-αSyn, we examined the amounts of hp-tau and p-αSyn that accumulated in 10 regions in rTg4510 mice fed the standard or doxycycline diet using immunohistochemistry: the hippocampal CA1 and CA3 areas, dentate gyrus, motor area, somatosensory area, entorhinal cortex, piriform cortex, amygdala, striatum, and substantia nigra. rTg4510 mice fed the doxycycline diet showed a significant decrease of hp-tau and p-αSyn deposition in the hippocampal CA1 and CA3 areas, motor area, somatosensory area, entorhinal cortex, piriform cortex, amygdala, and striatum, as compared to rTg4510 mice fed the standard diet (Fig. 2a, b). However, in the dentate gyrus and substantia nigra, where few hp-tau inclusions were observed, p-αSyn aggregates were scarce in rTg4510 mice fed the standard and doxycycline diet. As shown in the scatterplots, a positive correlation was found between the average amounts of hp-tau and p-αSyn that accumulated in each region (*r* = 0.85, *p* < 0.01) (Fig. 2c). Furthermore, positive correlations were observed between the amounts of hp-tau and p-αSyn that accumulated in each area (hippocampal CA1, *r* = 0.80, *p* < 0.01; hippocampal CA3, *r* = 0.94, *p* < 0.01; motor cortex, *r* = 0.74, *p* < 0.01; entorhinal cortex, *r* = 0.77, *p* < 0.01; piriform cortex, *r* = 0.88, *p* < 0.01; amygdala, *r* = 0.86, *p* < 0.01) (Fig. 2d-i). There was no amyloid β pathology in the brains of any mice (data not shown).

### Detection of proteinase K-resistant αSyn in rTg4510 mice

**Fig. 3:**
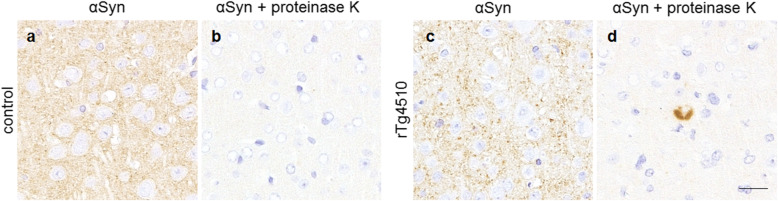
Immunohistochemistry for αSyn with the proteinase K treatment in the motor area of rTg4510 mice and control mice. The proteinase K treatment diminished neuropil staining in control mice (**a**, **b**) and rTg4510 mice (**c**, **d**), and a few proteinase K-resistant αSyn aggregates were found in rTg4510 mice (**d**). Scale bar: 20 μm (**a**-**d**)

To evaluate the proteinase K resistance of αSyn in rTg4510 mice, we performed immunohistochemistry for αSyn after proteinase K digestion. The immunoreactivity of endogenous αSyn in neuronal cells and neuropils was negligible in control mice after this treatment, whereas a few αSyn-positive dense, uniform aggregates were detected in the neuronal cytoplasm of rTg4510 mice (Fig. 3). These aggregates were found in the hippocampus, motor area, entorhinal cortex, piriform cortex and amygdala, in which excessive hp-tau and p-αSyn accumulation was observed, in rTg4510 mice (Supplementary Fig. 2). These aggregates were not detected without proteinase K digestion, and proteinase K-resistant αSyn-positive aggregates were morphologically different from p-αSyn-positive aggregates.

### Decreases in p-αSyn monomers and increases in ubiquitinated p-αSyn dimers in rTg4510 mice

**Fig. 4:**
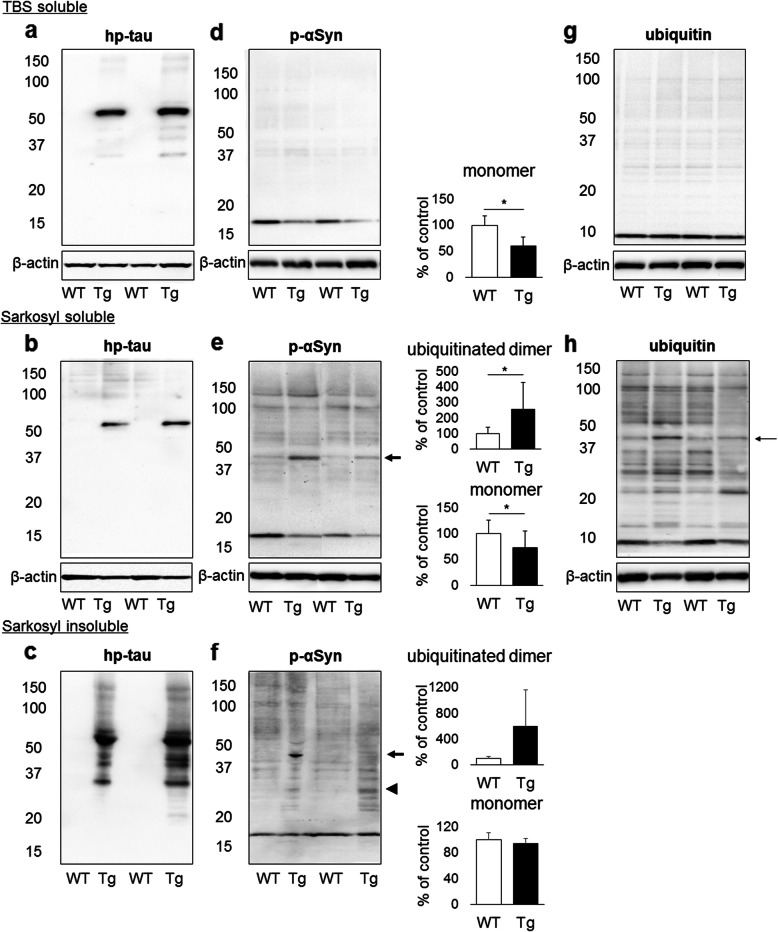
Western blotting analysis for hp-tau (AT8), p-αSyn (D1R1R) and ubiquitin in the TBS-soluble, sarkosyl-soluble, and sarkosyl-insoluble fractions from the hindbrain of rTg4510 mice (Tg) and control mice (WT) (*n* = 10 for TBS- and sarkosyl soluble fractions of Tg and WT mice, *n* = 3 for sarkosyl insoluble fractions of Tg and WT mice). A distinct band of hp-tau with a molecular weight of 50 kDa was detected in the TBS-soluble and sarkosyl-soluble fractions of Tg brains (**a**, **b**), and hp-tau was also detected in the sarkosyl-insoluble fractions of Tg brains (**c**). A single distinct band of p-αSyn monomers with a molecular weight of 17 kDa was detected in both mouse brains, and p-αSyn monomers were decreased in Tg brains (*p < 0.01, **d**). Additionally, p-αSyn oligomers were detected in the sarkosyl-soluble fractions of both mouse brains. p-αSyn monomers were decreased and ubiquitinated p-αSyn dimers with a molecular weight of 45 kDa (**arrow**) were increased in Tg brains (*p < 0.01, **e**). In addition to p-αSyn monomers, p-αSyn dimers (**arrowhead**) and ubiquitinated p-αSyn dimers (**arrow**) were detected in the sarkosyl-insoluble fractions of Tg brains (**f**). One distinct band of ubiquitin with a molecular weight of 10 kDa was detected in the TBS-soluble fractions of both mouse brains (**g**), and several bands of ubiquitinated proteins, including ubiquitinated p-αSyn dimers (**arrow**), were detected in the sarkosyl-soluble fractions of both mouse brains (**h**)

To detect αSyn oligomerization in the brain, protein samples in TBS-soluble, sarkosyl-soluble, and sarkosyl-insoluble fractions were examined by Western blotting. In each fraction, hp-tau-positive bands were detected in rTg4510 mice (Fig. 4a-c, Supplementary Fig. 4). In TBS-soluble fractions from rTg4510 and control mice, a p-αSyn-positive single band, corresponding to the monomeric form of αSyn, and a ubiquitin-positive single band, corresponding to the molecular weight of ubiquitin, were detected (Fig. 4d, g). The expression levels of the p-αSyn monomer were significantly lower in rTg4510 mice than in control mice (Fig. 4d). In sarkosyl-soluble fractions from rTg4510 and control mice, several bands corresponding to the monomeric and oligomeric forms of p-αSyn and several ubiquitin-positive bands corresponding to ubiquitin and ubiquitinated proteins were detected (Fig. 4e, h, Supplementary Fig. 5). The expression levels of the p-αSyn monomer were significantly lower in rTg4510 mice than in control mice (Fig. 4e). Furthermore, the expression levels of p-αSyn with a molecular weight of approximately 45 kDa were significantly higher in rTg4510 mice than in control mice. This 45 kDa band was also positive for ubiquitin, indicating ubiquitinated p-αSyn dimers (Fig. 4e, h). In sarkosyl-insoluble fractions from rTg4510 and control mice, several p-αSyn-positive bands, corresponding to the monomeric and oligomeric forms of αSyn were detected (Fig. 4f). Furthermore, in rTg4510 mice two distinct p-αSyn-positive bands were detected, approximately 30 and 45 kDa, indicating p-αSyn dimers and ubiquitinated p-αSyn dimers, respectively (Fig. 4f).

### αSyn phosphorylation enzyme in rTg4510 mice

**Fig. 5:**
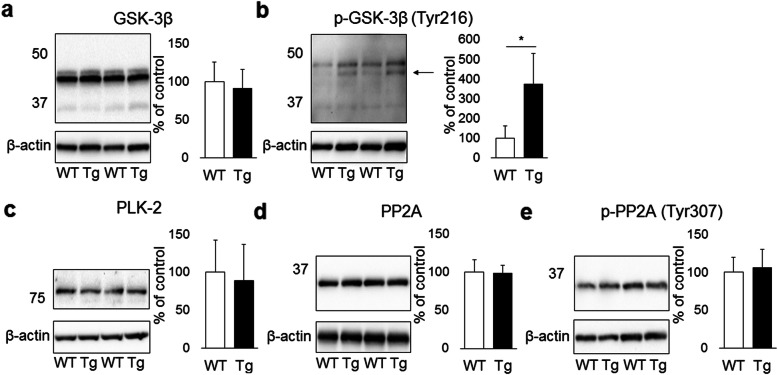
Western blotting analysis of kinases and phosphatase in TBS-soluble fractions from the hindbrain of rTg4510 mice (Tg, n = 10) and control mice (WT, n = 10). A significant increase was observed in p-GSK-3β (Tyr216) (lower band, **arrow**, *p < 0.01, **b**) in Tg brains, whereas no changes were noted in GSK-3β (**a**), PLK-2 (**c**), PP2A (**d**), and p-PP2A (Tyr307) (**e**)

The phosphorylation of αSyn is determined by the balance between the activities of kinases and phosphatases. To elucidate the mechanisms underlying changes in αSyn phosphorylation in rTg4510 mice, the levels of the major αSyn kinases (GSK-3β and PLK2) and phosphatase (PP2A) were quantified by Western blotting. No significant changes were observed in GSK-3β levels in rTg4510 mice (Fig. 5a). Western blotting for GSK-3β phosphorylated at Tyr216 (p-GSK-3β (Tyr216)), an activated form of GSK-3β, detected two distinct bands with molecular weights of approximately 46 kDa, corresponding to GSK-3β, and 48 kDa, corresponding to GSK-3α. The expression levels of p-GSK-3β (Tyr216) were higher in rTg4510 mice than in control mice (Fig. 5b). No significant differences were observed in the levels of PLK2, PP2A, or PP2A phosphorylated at Tyr307 (p-PP2A (Tyr307)), an inactivated form of PP2A, between rTg4510 mice and control mice (Fig. 5c-e).

### Spatial and temporal activation of GSK-3β in rTg4510 mice

**Fig. 6:**
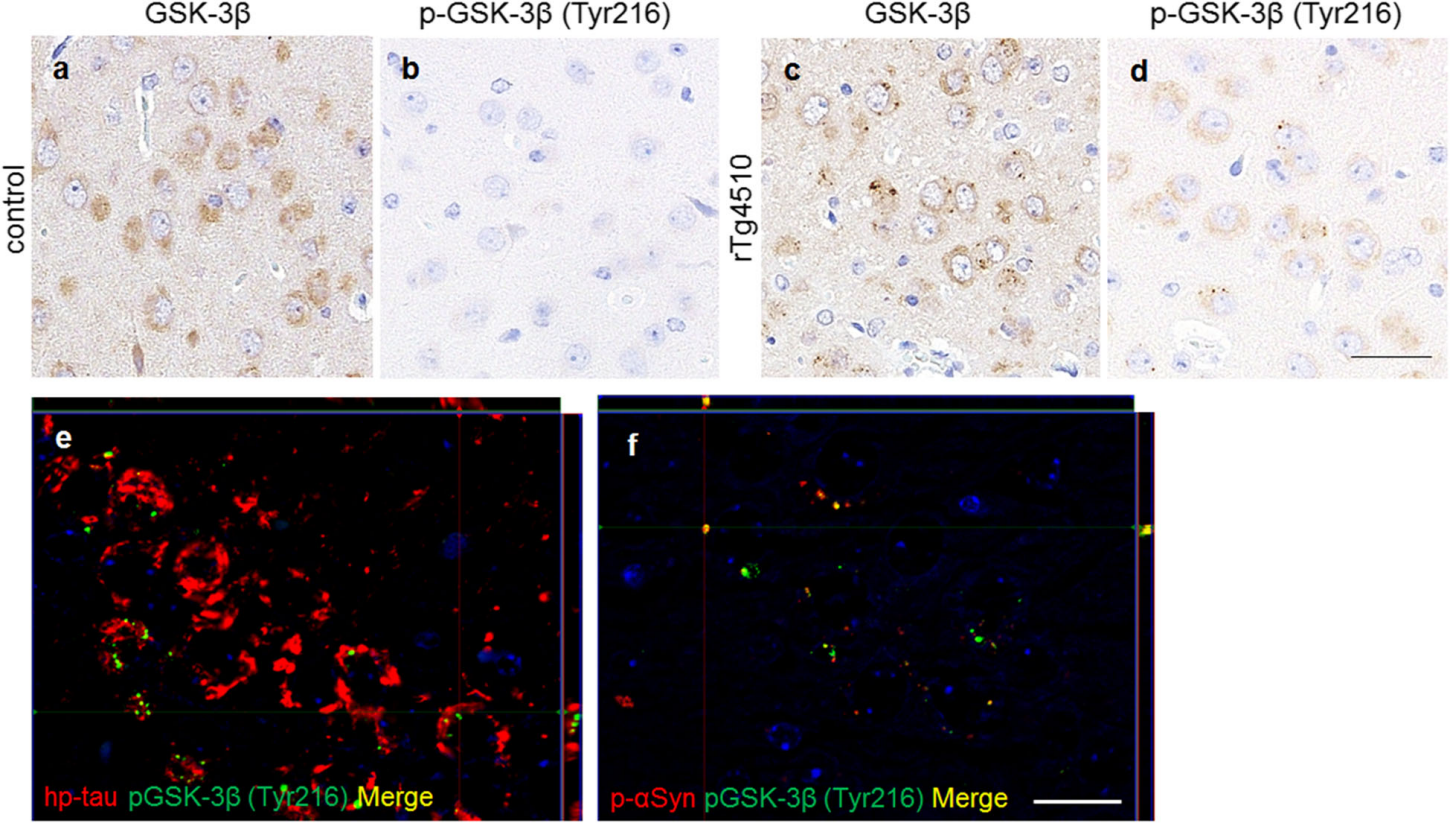
Immunohistochemistry for GSK-3β in the amygdala of rTg4510 and control mice. The cytoplasmic uniform distribution of GSK-3β was found in both mice (**a**, **c**). GSK-3β-positive grains were found in rTg4510 mice (**c**). P-GSK-3β (Tyr216)-positive cells were found in rTg4510 mice (**d**), but not in control mice (**b**). Double-labeling immunofluorescence in the hippocampus of rTg4510 mice revealed that p-GSK-3β (Tyr216)-positive grains were localized in hp-tau-positive cells (**e**). The majority of p-GSK-3β (Tyr216) grains did not co-localize with hp-tau aggregates (**e**). P-αSyn-positive aggregates localized in p-GSK-3β (Tyr216)-positive cells (**f**). p-αSyn-positive aggregates co-localized with p-GSK-3β (Tyr216)-positive grains (**f**). The reactions to hp-tau (**e**) and p-αSyn (**f**) are shown in red, and those to p-GSK-3β (Tyr216) (**e**, **f**) in green. Scale bars: 20 μm (**a**-**d**); 50 μm (**e**, **f**)

The immunohistochemical analysis revealed uniform GSK-3β expression in the neuronal cytoplasm of both rTg4510 and control mice, and GSK-3β-positive grains were also found in rTg4510 mice, but not in control mice (Fig. 6a, c). Furthermore, rTg4510 mice developed p-GSK-3β (Tyr216)-positive grains in the hippocampal CA1 and CA3 areas, motor area, somatosensory area, entorhinal cortex, piriform cortex, amygdala, and striatum, which corresponded to the distribution of hp-tau and p-αSyn aggregates in rTg4510 mice, but not in control mice (Fig. 6b, d, Supplementary Fig. 3). A double-labeling immunofluorescence analysis revealed that p-GSK-3β (Tyr216)-positive grains and hp-tau and p-αSyn aggregates were detected within the same cells (Fig. 6e, f). Moreover, p-αSyn aggregates were only observed in cells with hp-tau aggregates and p-GSK-3β (Tyr216)-positive grains, and p-GSK-3β (Tyr216)-positive grains were only detected in cells with hp-tau aggregates. Furthermore, the majority of p-GSK-3β (Tyr216)-positive grains did not co-localize with hp-tau aggregates (Fig. 6e), while most p-αSyn aggregates co-localized with p-GSK-3β (Tyr216)-positive grains (Fig. 6f).

### Distribution of activated GSK-3β in rTg4510 mice

**Fig. 7:**
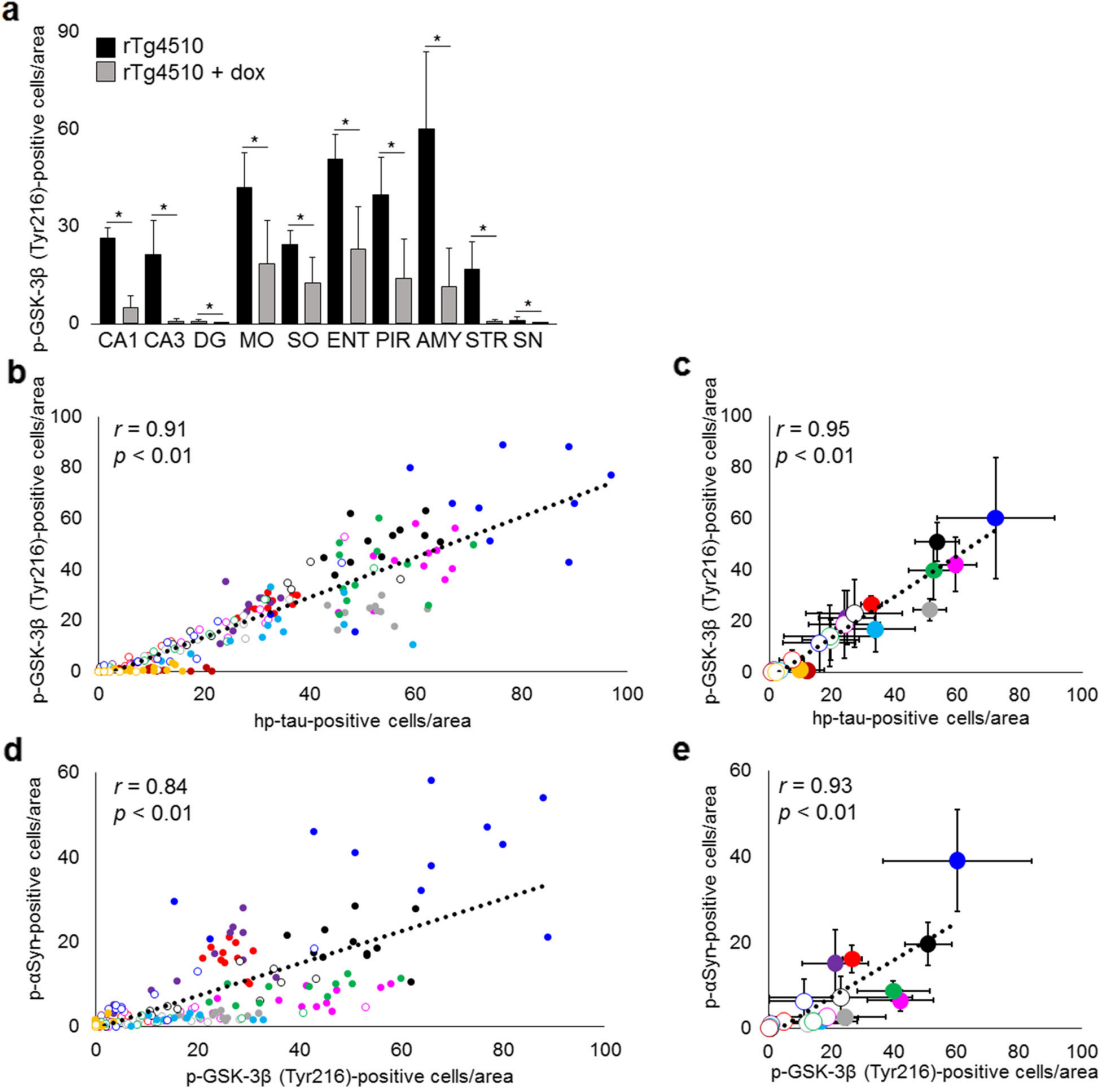
Semi-quantitative analysis of p-GSK-3β (Tyr216) in 10 regions: hippocampal CA1 and CA3 areas, dentate gyrus (DG), motor area (MO), somatosensory area (SO), entorhinal cortex (ENT), piriform cortex (PIR), amygdala (AMY), striatum (STR), and substantia nigra (SN), in rTg4510 mice fed the standard (*n* = 11) or doxycycline (dox) diet (n = 10). The accumulation of p-GSK-3β (Tyr216) was decreased in all regions studied in rTg4510 mice fed the dox diet (*p < 0.01, **a**). Positive correlations were observed between the number of hp-tau-positive cells and p-GSK-3β (Tyr216)-positive cells (*r* = 0.91, *p* < 0.01, **b**) and between the average number of hp-tau-positive cells and p-GSK-3β (Tyr216)-positive cells in each region (*r* = 0.95, *p* < 0.01, **c**). Moreover, positive correlations were found between the number of p-αSyn-positive cells and p-GSK-3β (Tyr216)-positive cells (*r* = 0.84, *p* < 0.01, **d**) and between the average number of p-αSyn-positive cells and p-GSK-3β (Tyr216)-positive cells in each region (*r* = 0.93, *p* < 0.01, **e**). Colors: red, CA1; purple, CA3; deep red, DG; pink, MO; gray; SO; black, ENT; green; PIR; blue, AMY; light blue, STR; orange, SN. Diagrams: circular dot, rTg4510 mice fed the control diet; circle, rTg4510 mice fed the dox diet

To evaluate the relationship between the distribution of activated GSK-3β and hp-tau or p-αSyn deposition, we examined the number of p-GSK-3β (Tyr216)-positive cells using immunohistochemistry in rTg4510 mice fed the standard or doxycycline diet. Numerous p-GSK-3β (Tyr216)-positive cells were found in the hippocampal CA1 area, motor area, somatosensory area, entorhinal cortex, piriform cortex, amygdala, and striatum, in which hp-tau and p-αSyn accumulation was excessive (Fig. 7a). However, few p-GSK-3β (Tyr216)-positive cells were observed in the dentate gyrus and substantia nigra. As shown in the scatterplots, positive correlations were observed between the number of hp-tau-positive cells and p-GSK-3β (Tyr216)-positive cells (*r* = 0.91, *p* < 0.01), and between the average number of hp-tau-positive cells and p-GSK-3β (Tyr216)-positive cells in each area (*r* = 0.95, *p* < 0.01) (Fig. 7b, c). Furthermore, positive correlations were noted between the number of p-GSK-3β (Tyr216)-positive cells and p-αSyn-positive cells (*r* = 0.84, *p* < 0.01) and between the average number of p-GSK-3β (Tyr216)-positive cells and p-αSyn-positive cells in each area (*r* = 0.93, *p* < 0.01) (Fig. 7d, e).

## Discussion

We previously reported that rTg4510 mice developed not only hp-tau aggregates, but also p-αSyn aggregates in their brains, and the accumulation of both of these proteins increased in an age-dependent manner [52]. We also detected hp-tau and p-αSyn aggregates in aged rTg4510 mice fed a doxycycline diet, and confirmed that the accumulation of p-αSyn in rTg4510 mice treated with doxycycline, which reduced pathological tau levels, was less than that in untreated rTg4510 mice. Tau accumulation, even at a very low concentration, results in the co-accumulation of αSyn polymers in vitro, and this is strongly enhanced in a concentration-dependent manner [15]. Furthermore, the co-expression of tau with αSyn leads to small αSyn aggregates with enhanced toxicity, which drives dendritic and synaptic damage in vitro [15]. In addition to rTg4510 mice, the accumulation of αSyn has also been found in DLB-AD transgenic mice, which express human mutant APP, PSEN1, tau (P301L), and αSyn (A53T) [5], and in bigenic mice that express wild-type human αSyn and mutant tau (P301L) [15]. In rTg4510 mice, transgene suppression with doxycycline was shown to lower tau production by 85% from untreated levels [48] and reduce neuronal death and neuroinflammation [8]. In the present study, positive correlations were observed between hp-tau and p-αSyn accumulation in all regions studied in rTg4510 mice fed the control and doxycycline diets. Therefore, the present results suggest that hp-tau promotes αSyn phosphorylation and accumulation in rTg4510 mice, and the accumulation of hp-tau increases that of p-αSyn in a load-dependent manner in vivo.

Proteinase K resistance is a salient feature of the misfolded proteins relevant to prion diseases [11]. Additionally, proteinase K-resistant αSyn aggregates are found in human α-synucleinopathies, and proteinase K treatments are useful for detecting the cytoplasmic and neuritic pathologies of these patients [14, 38]. Furthermore, aggregated αSyn in the LBs and neurites of PD and DLB are phosphorylated [37]. In the present study, we detected a few proteinase K-resistant αSyn-positive dense, uniform aggregates in areas with excessive hp-tau accumulation in rTg4510 mice, and these were morphologically different from p-αSyn granular deposits. Some α-synucleinopathy model mice, such as A53T and A30P αSyn transgenic mice, show the deposition of proteinase K-resistant αSyn [37, 53]. A53T αSyn transgenic mice developed proteinase K-resistant αSyn deposits in the presynapses. These deposits were composed of non-phosphorylated αSyn, while cytoplasmic phosphorylated LB-like inclusions were not resistant to proteinase K [53]. A30P αSyn transgenic mice develop proteinase K-resistant LB-like αSyn-positive structures in the neuronal somata and neurites, predominantly in the brain stem and spinal cord [37]. In the present study, morphological differences between proteinase K-resistant αSyn and p-αSyn deposits suggested that proteinase K-resistant αSyn was not phosphorylated. Furthermore, the similar regional distribution of hp-tau and proteinase K-resistant αSyn suggested that αSyn gained proteinase K resistance in association with hp-tau accumulation.

The oligomeric form of αSyn is considered to play a central role in the pathogenesis of PD and other α-synucleinopathies [43]. The phosphorylation of αSyn leads to oligomerization [21] and is the major underlying process for the formation of LBs [57]. In the present study, we detected decreases in p-αSyn monomers in TBS- and sarkosyl-soluble fractions and increases in ubiquitinated p-αSyn dimers in sarkosyl-soluble and insoluble fractions in rTg4510 mice. These results suggested that p-αSyn was more likely to form insoluble oligomers in the brains of rTg4510 mice. An in vitro study showed that the co-incubation of tau with αSyn, even at very low concentrations of tau, resulted in the accumulation of high-molecular-weight αSyn [15]. Tau oligomers, in conjunction with αSyn oligomers, enhance their cytotoxicity and accelerate the formation of αSyn inclusions, suggesting that toxic protein cross-seeding and oligomerization are the pathognomic factors involved in neurodegenerative diseases [4, 22]. αSyn with mutations of familial PD, such as A53T, A30P, and E46K, show a faster rate of protein aggregation and greater propensity to self-interact and form dimeric structures than wild-type αSyn [44]. Furthermore, αSyn dimers are important for mature fibril formation because αSyn dimerization accelerates the formation of neurotoxic intermediate aggregate species and amyloid fibrils [47]. We previously demonstrated that in addition to p-αSyn-positive grains, rTg4510 mice developed p-αSyn-positive spherical LB-like inclusions [51]. Based on these findings, we speculate that p-αSyn-positive grains are associated with the cross-seeding of hp-tau and αSyn, while p-αSyn-positive spherical LB-like inclusions are also associated with the self-aggregation of p-αSyn.

Several kinases and phosphatases, including GSK-3β, PLK2, and PP2A, are associated with the phosphorylation of tau and α-Syn [10, 27, 35, 55]. In AD, the most prevalent age-related neurodegenerative disorder, amyloid β has been suggested to induce the activation of GSK-3β by inhibiting Wnt signaling, and the activation of GSK-3β is closely associated with amyloid β deposition [40, 45]. In the present study, we detected the activation of GSK-3β in rTg4510 mice fed the control and doxycycline diets, and a positive correlation was observed between hp-tau accumulation and GSK-3β activation in rTg4510 mice, whereas amyloid β deposits were absent. Therefore, hp-tau accumulation may have induced the activation of GSK-3β in rTg4510 mice independently from Wnt signaling and amyloid β deposition. The activation of PLK2 and inactivation of PP2A have been demonstrated in a rat model injected with a lentivirus harboring human wild-type or P301L mutant tau [26]. Moreover, rTg4510 mice show a decrease in PP2A A subunit levels, a structural subunit, but no changes in the levels of the PP2A B subunit, a regulatory subunit, the PP2A C subunit, a catalytic subunit responsible for enzyme activity, or GSK-3β [36]. In the present study, we detected an increase in activated GSK-3β levels with no changes in GSK-3β levels, and no changes in PLK2, PP2A C subunit, or inactivated PP2A levels in rTg4510 mice. These results suggest that hp-tau accumulation induces the activation of GSK-3β in vivo. The difference in PLK2 and PP2A activities between rTg4510 mice and the lentivirus-injected rat model may be due to the expression levels of hp-tau, the promoter that was used, or species differences between mice and rats.

In addition to tau, GSK-3β phosphorylates αSyn, which may enhance αSyn aggregation and neurotoxicity [57]. In the present study, a positive correlation was observed between GSK-3β activation and p-αSyn accumulation in rTg4510 mice. Previous studies reported that the overactivation of GSK-3β led to excessive p-αSyn accumulation in vivo [6, 16]. Furthermore, the up-regulation of Wnt signaling via the overexpression of β-catenin or inhibition of GSK-3β was found to protect PD models from excessive p-αSyn accumulation or the development of motor deficits [51, 59]. Regarding cross-seeding, in which the seeds of one protein may induce the aggregation of another protein, previous studies suggested direct interactions between tau and αSyn [31]. In rTg4510 mice, the majority of p-αSyn-positive grains and p-αSyn-positive spherical LB-like inclusions did not co-localize with hp-tau aggregates [52]. Therefore, we speculate that in addition to direct interactions between both proteins, the activation of GSK-3β is of importance for the accumulation of p-αSyn in rTg4510 mice.

Recent findings support αSyn, directly and via GSK-3β, promoting tau phosphorylation and aggregation. αSyn binds to tau via the microtubule-binding region of tau and serves as a cofactor to promote hp-tau oligomerization [7, 15, 23]. Furthermore, αSyn oligomers and protofibrils inhibit the formation of tau-mediated microtubule assembly, leading to the hyperphosphorylation and aggregation of tau [41]. The overexpression of αSyn increases GSK-3β activity and leads to hp-tau accumulation, and αSyn, hp-tau, and p-GSK-3β (Tyr216) were found to co-localize in large inclusion bodies [9, 18, 56]. Moreover, A53T and A30P αSyn transgenic mice and αSyn-injected wild-type mice accumulated hp-tau in their brains [13, 25, 56]. Therefore, an intracellular positive feedback loop between hp-tau and p-αSyn via the promotion of oligomerization with each other directly and/or through the activation GSK-3β has been suggested [40]. In the present study, we confirmed that the overexpression of human P301L mutant tau promoted the activation of GSK-3β, and induced the phosphorylation, proteinase K resistance, oligomerization, insolubilization, and accumulation of endogenous mouse αSyn. Collectively, previous findings and the present results indicate that hp-tau and p-αSyn accelerate phosphorylation and aggregation with each other via the activation of GSK-3β and exacerbate the pathology in rTg4510 mice. These results highlight the importance of this cellular synergic effect between hp-tau and p-αSyn in neurodegenerative disorders.

## Supplementary information

**Additional file 1: Supplemental Figure 1.** Immunohistochemistry (IHC) with hp-tau (AT8) and p-αSyn (D1R1R) and without primary antibodies (control) in rTg4510 mice fed the control diet. Hp-tau and p-αSyn-positive aggregates were found in the hippocampal CA1 (**a**, **b**) and CA3 (**d**, **e**) areas, motor area (**g**, **h**), somatosensory area (**j**, **k**), entorhinal cortex (**m**, **n**), piriform cortex (**p**, **q**), and striatum (**s**, **t**). IHC without a primary antibody did not detect any deposits in the brain (**c**, **f**, **i**, **l**, **o**, **r**, **u**). Scale bar: 20 μm (**a**-**u**).

**Additional file 2: Supplemental Figure 2.** Immunohistochemistry for αSyn with the proteinase K treatment in rTg4510 mice. rTg4510 mice developed proteinase K-resistant αSyn in the hippocampus (**a**), entorhinal cortex (**b**), piriform cortex (**c**), and amygdala (**d**). Scale bar: 20 μm (**a**-**d**).

**Additional file 3: Supplemental Figure 3** Immunohistochemistry for p-GSK-3β (Tyr216) in rTg4510 mice. P-GSK-3β (Tyr216)-positive cells were found in the hippocampal CA1 (**a**) and CA3 (**b**) areas, motor area (**c**), somatosensory area (**d**), entorhinal cortex (**e**), piriform cortex (**f**), and striatum (**g**). Scale bar: 20 μm (**a**-**g**).

**Additional file 4: Supplementary Figure 4.** Western blotting analysis of TBS-soluble, sarkosyl-soluble, and sarkosyl-insoluble fractions from the hindbrain of rTg4510 mice (Tg) and control mice (WT) (*n* = 10 for TBS- and sarkosyl soluble fractions of Tg and WT mice, *n* = 3 for sarkosyl insoluble fractions of Tg and WT mice). The expression levels of total hp-tau (AT8) were increased in the TBS- (**a**) and sarkosyl-soluble (**b**) and sarkosyl-insoluble fractions (**c**) of rTg4510 mice.

**Additional file 5: Supplementary Figure 5.** Western blotting analysis of sarkosyl-soluble fractions from the hindbrain of rTg4510 mice (Tg) and control mice (WT) using anti-p-αSyn antibody 81A (**a**) and #64 (**b**). P-αSyn oligomers including ubiquitinated dimers (**arrow**) were detected in Tg and WT mice.
