# Supplementary figures and images for: Phosphorylation and oligomerization of α-synuclein associated with GSK-3β activation in the rTg4510 mouse model of tauopathy
Source: Acta Neuropathol Commun. 2020 Jun 19;8:86. doi: 10.1186/s40478-020-00969-8 (PMC7304163; doi:10.1186/s40478-020-00969-8)

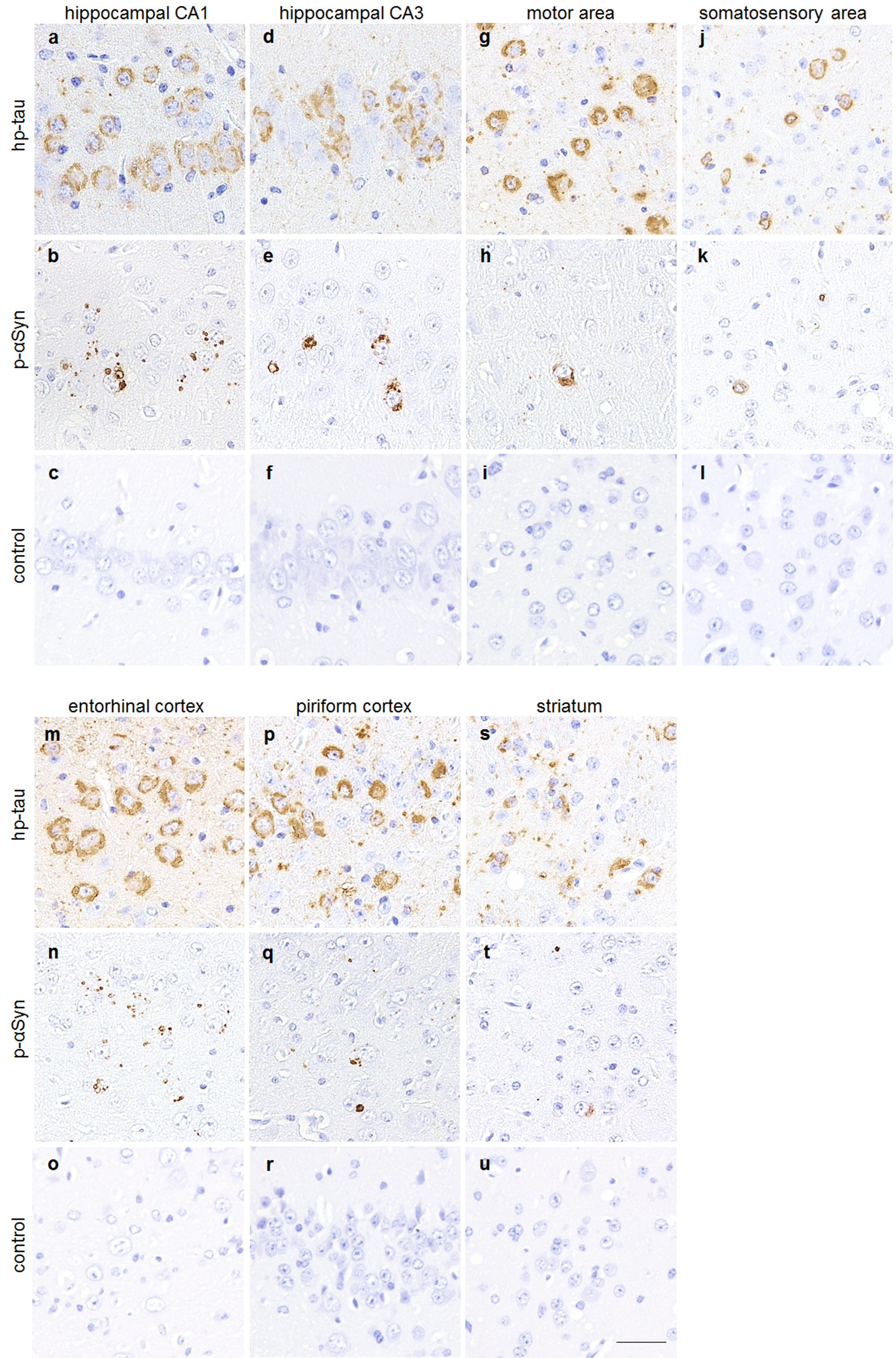

Supplement: Supplementary file 1 — Additional file 1: Supplemental Figure 1. Immunohistochemistry (IHC) with hp-tau (AT8) and p-αSyn (D1R1R) and without primary antibodies (control) in rTg4510 mice fed the control diet. Hp-tau and p-αSyn-positive aggregates were found in the hippocampal CA1 (a, b) and CA3 (d, e) areas, motor area (g, h), somatosensory area (j, k), entorhinal cortex (m, n), piriform cortex (p, q), and striatum (s, t). IHC without a primary antibody did not detect any deposits in the brain (c, f, i, l, o, r, u). Scale bar: 20 μm (a-u). [file 40478_2020_969_MOESM1_ESM.tif]

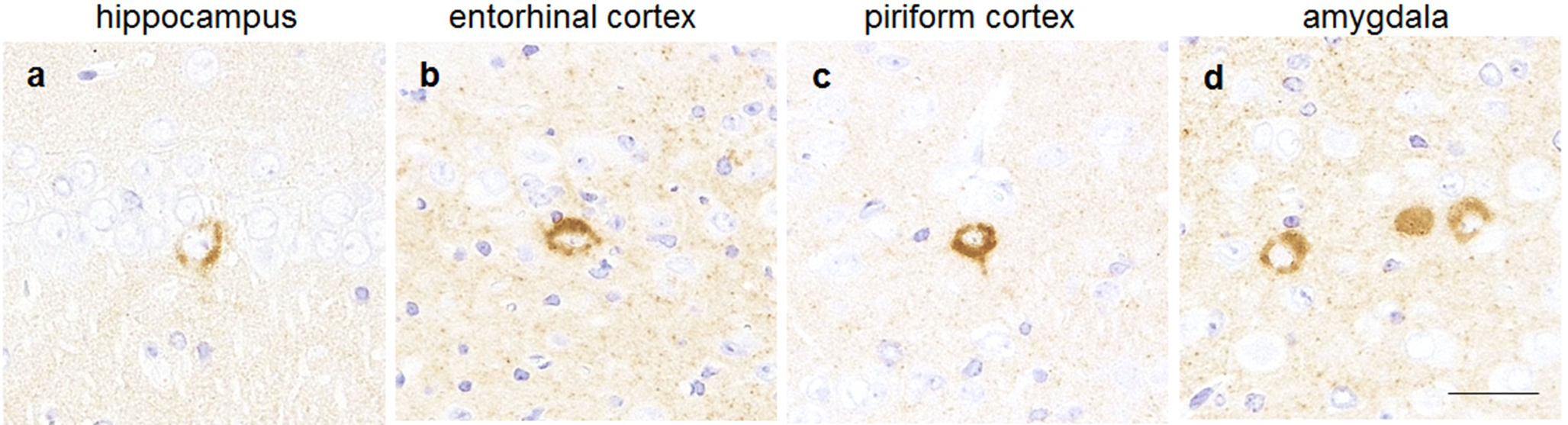

Supplement: Supplementary file 2 — Additional file 2: Supplemental Figure 2. Immunohistochemistry for αSyn with the proteinase K treatment in rTg4510 mice. rTg4510 mice developed proteinase K-resistant αSyn in the hippocampus (a), entorhinal cortex (b), piriform cortex (c), and amygdala (d). Scale bar: 20 μm (a-d). [file 40478_2020_969_MOESM2_ESM.tif]

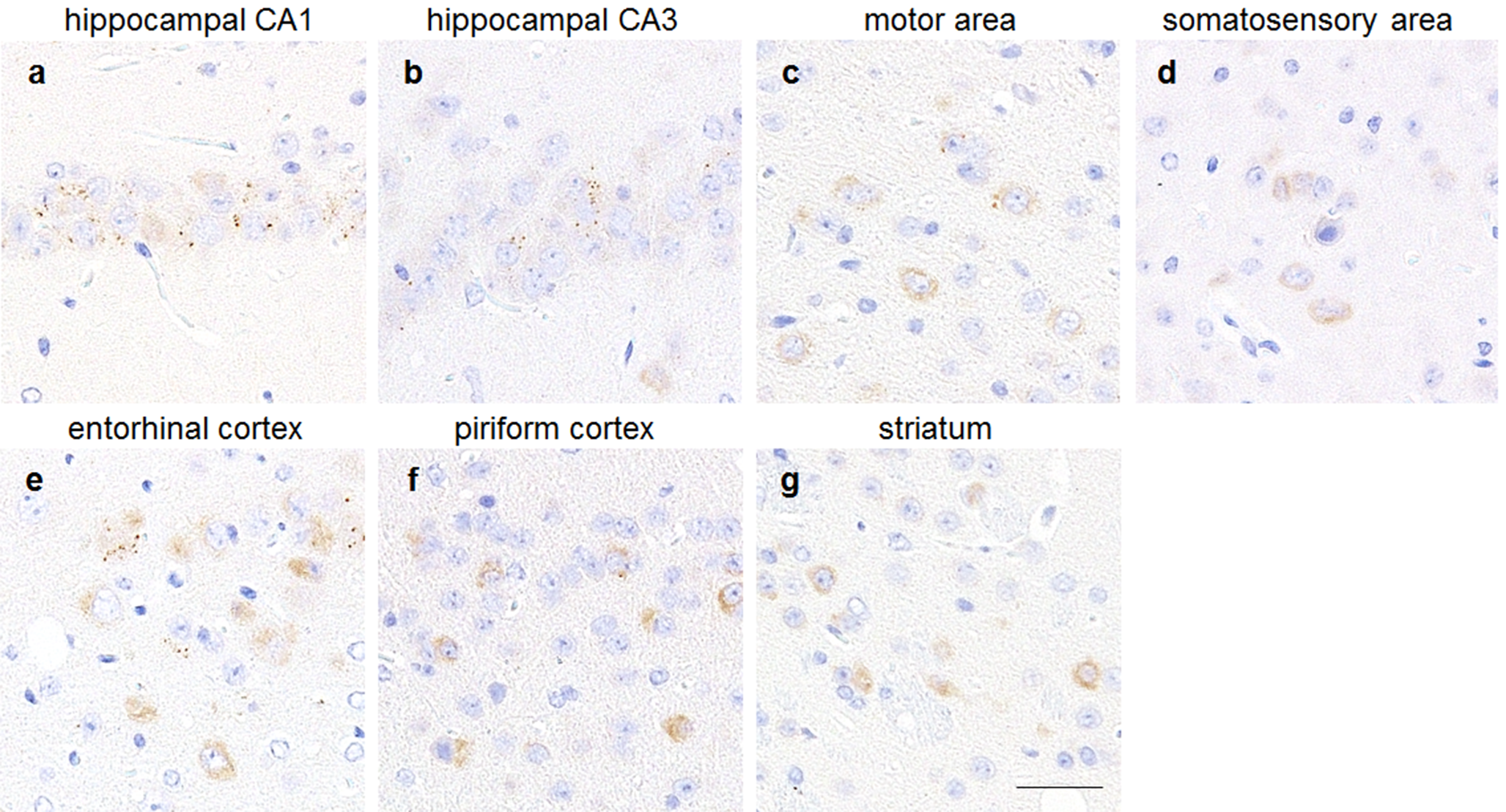

Supplement: Supplementary file 3 — Additional file 3: Supplemental Figure 3 Immunohistochemistry for p-GSK-3β (Tyr216) in rTg4510 mice. P-GSK-3β (Tyr216)-positive cells were found in the hippocampal CA1 (a) and CA3 (b) areas, motor area (c), somatosensory area (d), entorhinal cortex (e), piriform cortex (f), and striatum (g). Scale bar: 20 μm (a-g). [file 40478_2020_969_MOESM3_ESM.tif]

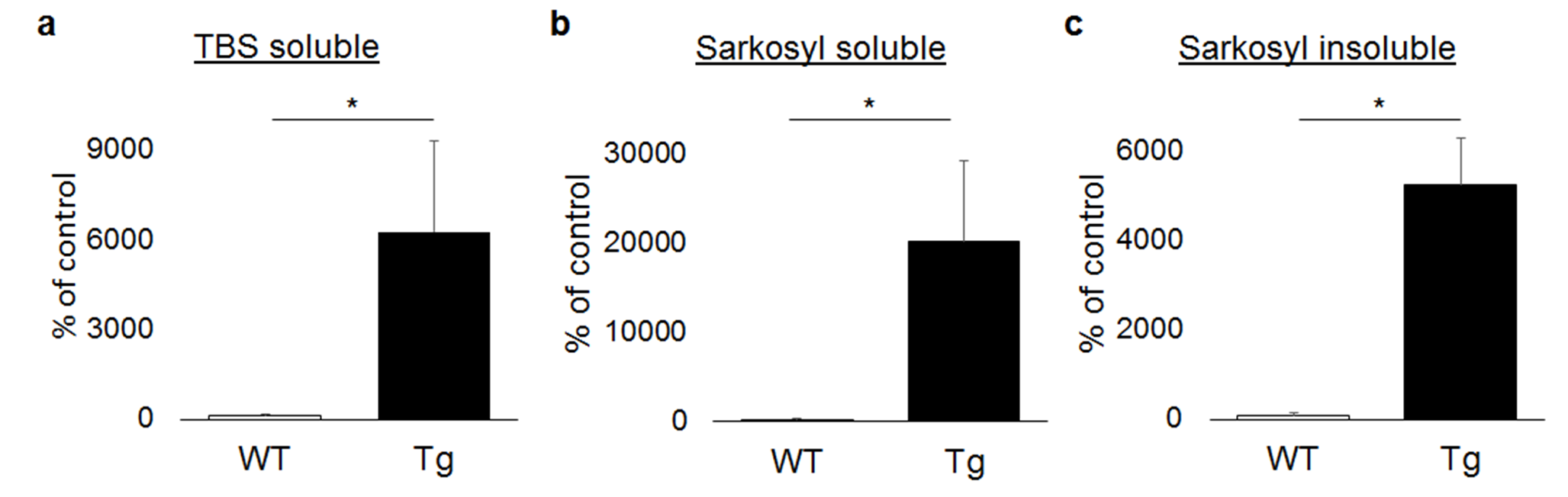

Supplement: Supplementary file 4 — Additional file 4: Supplementary Figure 4. Western blotting analysis of TBS-soluble, sarkosyl-soluble, and sarkosyl-insoluble fractions from the hindbrain of rTg4510 mice (Tg) and control mice (WT) (n = 10 for TBS- and sarkosyl soluble fractions of Tg and WT mice, n = 3 for sarkosyl insoluble fractions of Tg and WT mice). The expression levels of total hp-tau (AT8) were increased in the TBS- (a) and sarkosyl-soluble (b) and sarkosyl-insoluble fractions (c) of rTg4510 mice. [file 40478_2020_969_MOESM4_ESM.tif]

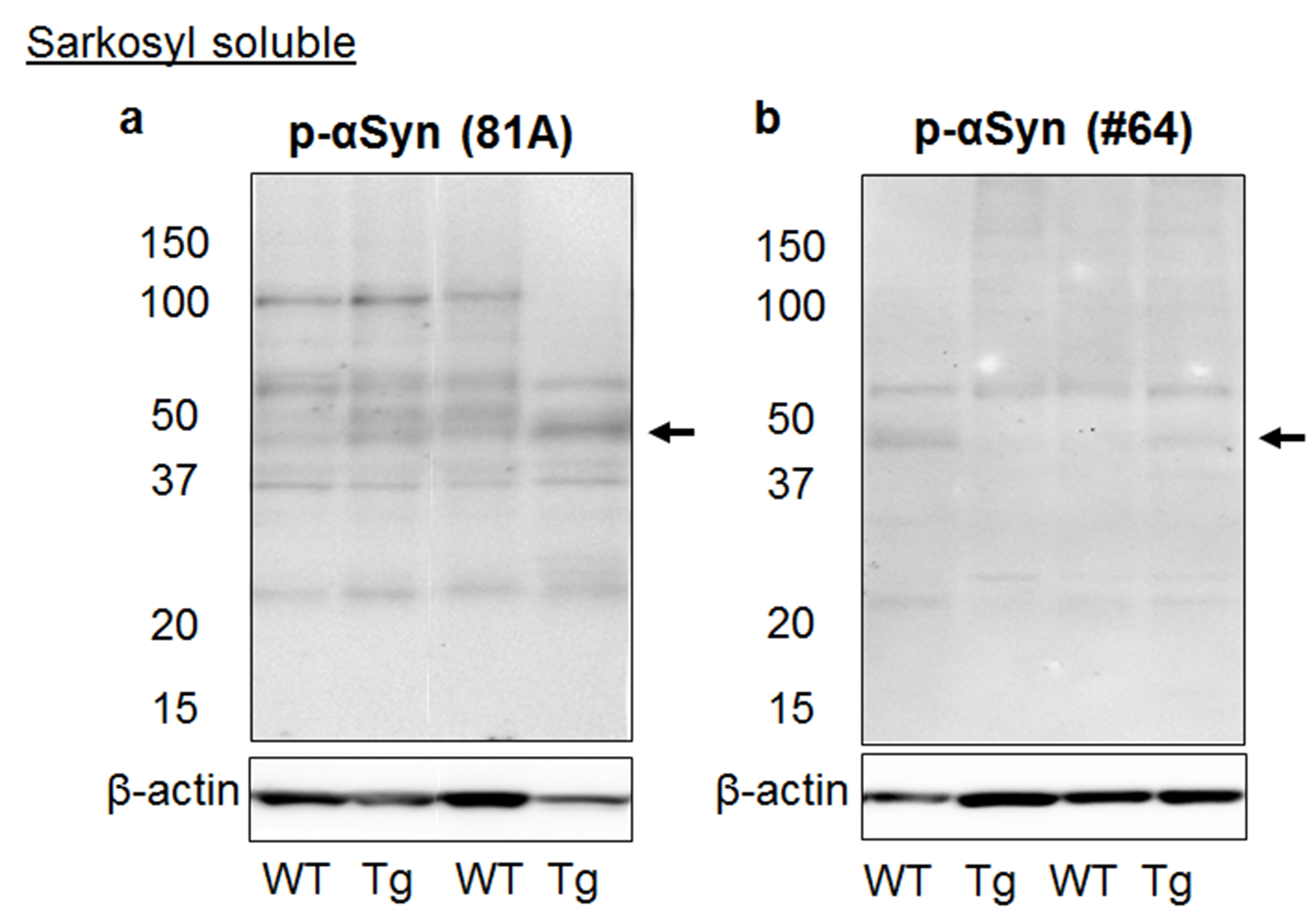

Supplement: Supplementary file 5 — Additional file 5: Supplementary Figure 5. Western blotting analysis of sarkosyl-soluble fractions from the hindbrain of rTg4510 mice (Tg) and control mice (WT) using anti-p-αSyn antibody 81A (a) and #64 (b). P-αSyn oligomers including ubiquitinated dimers (arrow) were detected in Tg and WT mice. [file 40478_2020_969_MOESM5_ESM.tif]
